# Does Preliminary Chest Shape Assessment Improve the Prognostic Risk Stratification of Individuals with Mitral Annular Disjunction? A Case Report and Narrative Review

**DOI:** 10.3390/jcm14072277

**Published:** 2025-03-26

**Authors:** Andrea Sonaglioni, Gian Luigi Nicolosi, Giovanna Elsa Ute Muti-Schünemann, Gaetana Anna Rispoli, Michele Lombardo, Paola Muti

**Affiliations:** 1 Division of Cardiology, IRCCS MultiMedica, 20123 Milan, Italy; michele.lombardo@multimedica.it; 2 Division of Cardiology, Policlinico San Giorgio, 33170 Pordenone, Italy; gianluigi.nicolosi@gmail.com; 3 Department of Emergency, Fondazione IRCSS Ca’ Granda, Ospedale Maggiore Policlinico, 20122 Milan, Italy; giovanna.muti@unimi.it; 4 Division of Radiology, IRCCS MultiMedica, 20123 Milan, Italy; gaetanaanna.rispoli@multimedica.it; 5 Department of Biomedical, Surgical and Dental Sciences, University of Milan, 20122 Milan, Italy; pmuti26@gmail.com; 6 IRCCS MultiMedica, 20138 Milan, Italy

**Keywords:** mitral annular disjunction, mitral valve prolapse, multimodality imaging assessment, arrhythmic burden, modified Haller index

## Abstract

**Background:** Mitral annular disjunction (MAD), a mitral annular abnormality involving the whole mitral valve annulus circumference, commonly detected in individuals with mitral valve prolapse (MVP), has been recently recognized as a potential risk factor for malignant ventricular arrhythmias (VAs) and sudden cardiac death. Recent evidence indicates that a multimodality imaging assessment comprehensive of echocardiography, cardiac magnetic resonance (CMR), and cardiac computed tomography angiography (CCTA) may improve MAD detection. To date, no previous author has considered the potential influence of chest wall conformation on MAD presence. Considering the strong association between MVP and anterior chest wall deformities and the increased prevalence of MAD among MVP individuals, we have hypothesized that MAD presence might be more frequently detected among MVP individuals with a narrow anteroposterior (A-P) thoracic diameter and/or concave-shaped chest wall conformation, as noninvasively assessed by the modified Haller index (MHI). **Methods:** Herein, we present a case of MVP female with relevant MAD distance and moderate mitral regurgitation (MR) who underwent a diagnostic study comprehensive of transthoracic echocardiography, transesophageal echocardiography, CMR, CCTA, and exercise stress echocardiography. **Results:** The patient was found with a concave-shaped chest wall conformation (MHI > 2.5) and narrow A-P thoracic diameter (<13.5 cm), with a moderate and non-hemodynamically significant MR, without areas of LGE on CMR and with low arrhythmic profile. **Conclusions:** A preliminary chest shape assessment by the MHI might improve the prognostic risk stratification of MVP patients with MAD, potentially identifying a benign phenotype of MVP individuals, i.e., those with a narrow A-P thoracic diameter.

## 1. Introduction

Mitral annular disjunction (MAD) is defined as the spatial displacement of the left atrial wall-mitral leaflet junction from the left ventricular (LV) wall during systole [1].

It is frequently detected in individuals with mitral valve prolapse (MVP), with a different prevalence depending on the specific imaging modality used for its identification and measurement [2,3]; in this regard, a recent systematic review including 23 studies conducted between 2005 and 2025, reported that the average MAD prevalence was 20% for the studies assessing MAD with cardiac computed tomography angiography (CCTA), 31.3% for those with transthoracic echocardiography (TTE), 44.7% for those with transesophageal echocardiography (TEE) and finally 47% for those with cardiac magnetic resonance (CMR) [4].

Even if imaging studies have reported a more frequent location of MAD at the level of P2 [5] or P3 scallop [6], pathological studies have described the MAD presence at any point around the mitral valve annulus (MVA) [7]. Accordingly, it is more appropriate to consider MAD as a circumferential phenomenon involving the entire MVA circumference [5,6].

Several studies [6,8,9,10,11] have demonstrated an association between MAD and the occurrence of premature ventricular complexes, ventricular arrhythmias (VAs), and, potentially, sudden cardiac death. It is hypothesized that MAD causes hypermobility of the mitral valve (MV) apparatus, leading to increased traction on the papillary muscles and the basal inferolateral LV wall. The continuous abnormal tugging on the sub-mitral apparatus can result in myocardial fibrosis, thus creating possible foci for VAs origin [12].

Multimodality imaging may play an important role in both the diagnosis and the prognostic risk stratification of MAD individuals. During the last two decades, a number of authors have evaluated MAD by using different imaging techniques, such as TTE [2,8,13,14,15,16,17,18,19,20,21,22,23], TEE [15,24], CMR [3,11,15,25,26,27,28], and cardiac computed tomography angiography (CCTA) [29]. The aforementioned imaging modalities have demonstrated a different diagnostic accuracy in assessing MAD presence and extent.

Our study group has recently demonstrated that among MVP individuals, those with MAD are more frequently found with a narrow anteroposterior (A-P) thoracic diameter due to various degrees of anterior chest wall deformity, as noninvasively assessed by the non-radiological modified Haller index (MHI) [30]. Based on our “mechanical theory”, the sternal compression on cardiac chambers might represent the main extrinsic determinant of the basal longitudinal strain attenuation detected in MVP individuals with MAD, potentially identifying a benign phenotype of MAD individuals without any intrinsic myocardial dysfunction [18].

As far as we know, the relationship between MAD and chest wall conformation has still not been thoroughly assessed in clinical practice.

Herein, we present a clinical case of a MAD female with a narrow A-P thoracic diameter referred to our cardiology outpatient clinic because of resting palpitations and exercise-induced dyspnea; she underwent a multimodality imaging assessment comprehensive of TTE, TEE, CMR, CCTA, and exercise stress echocardiography (ESE).

## 2. Clinical Case

A 62-year-old woman (BSA 1.69 m^2^, BMI 21.8 Kg/m^2^), affected by mild dyslipidemia in treatment with rosuvastatin 5 mg plus ezetimibe 10 mg, without a previous history of cardiovascular disease and without any non-cardiovascular comorbidity, was referred to our cardiology outpatient clinic to perform conventional TTE due to resting palpitations and exercise-induced dyspnea. A previous 24-h ECG Holter monitoring showed constant sinus rhythm with normal atrioventricular and intra-ventricular conduction and sporadic isolated VAs (approximately 250/24-h), occasionally perceived by the patient. She was prescribed bisoprolol 1.25 mg once daily.

**Figure 1:**
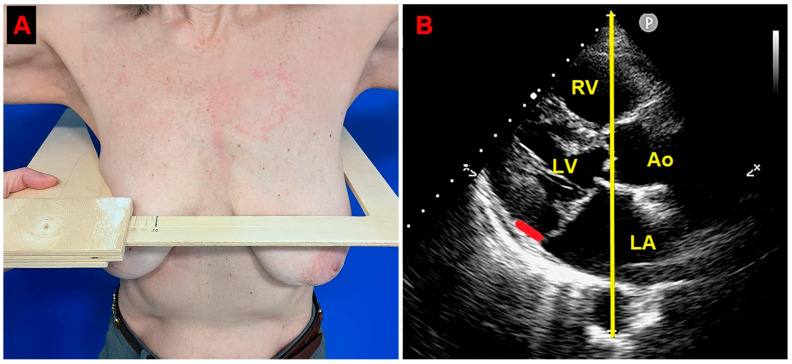
Modified Haller index, obtained by dividing the latero-lateral thoracic diameter by the anteroposterior thoracic diameter. (**A**) The latero-lateral thoracic diameter is measured by a rigid ruler coupled to a level. (**B**) The anteroposterior thoracic diameter is measured from the echocardiographic parasternal long-axis view as the distance between the true apex of the sector and the posterior wall of the descending aorta. The bold red line indicates the MAD distance. The bold yellow line indicates the anteroposterior thoracic diameter. Ao, aorta; LA; left atrium; LV, left ventricle; RV, right ventricle.

A preliminary chest shape assessment, as noninvasively assessed by the MHI [30], obtained by dividing the latero-lateral (L-L) thoracic diameter by the A-P thoracic diameter, revealed a concave-shaped chest wall conformation (L-L thoracic diameter = 29 cm, A-P thoracic diameter = 11 cm, estimated MHI = 2.6) (Figure 1).

**Figure 2:**
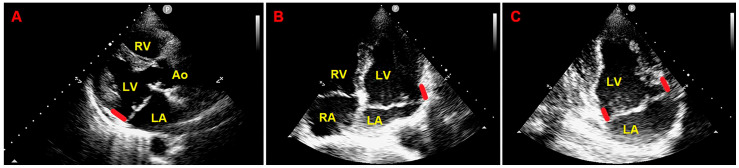
Transthoracic echocardiography. MAD assessment at end-systole from the parasternal long-axis view (**A**), from the apical four-chamber view (**B**), and from the apical two-chamber view (**C**). The bold red line indicates the MAD distance. Ao, aorta; LA; left atrium; LV, left ventricle; RA, right atrium; RV, right ventricle.

Resting TTE measurements revealed normal cardiac chambers cavity sizes (left ventricular end-diastolic diameter = 46 mm, right ventricular basal end-diastolic diameter = 33 mm, left atrial antero-posterior end-systolic diameter = 40 mm, right atrial longitudinal diameter 50 mm), grade 1 LV diastolic dysfunction and normal biventricular systolic function [left ventricular ejection fraction (LVEF) estimated with the biplane Simpson’s method = 60%; tricuspid annular plane systolic excursion (TAPSE) = 27 mm]; the aortic valve was tricuspid with normal function; a MVP with myxomatous degeneration of both leaflets and moderate mitral regurgitation (MR) was observed; a concomitant MAD was detected from the parasternal long-axis view (systolic infero-lateral MAD distance = 11 mm), from the apical four-chamber view (systolic antero-lateral MAD distance = 9 mm) and from the apical two-chamber view (systolic infero-medial MAD distance = 9 mm and systolic anterior MAD distance = 6 mm) (Figure 2); the estimated systolic pulmonary artery pressure (sPAP) was 30 mmHg.

**Figure 3:**
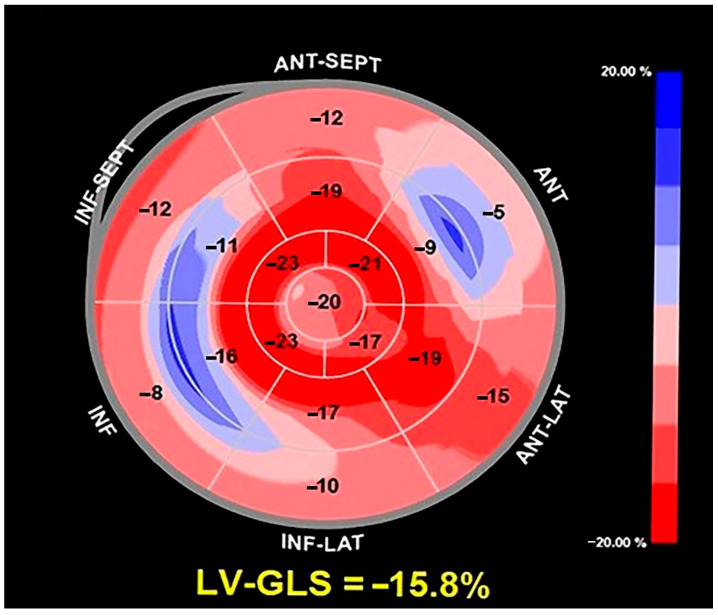
Bull’s-eye plot illustrating the regional longitudinal strain at basal, mid, and apical levels along with LV-GLS magnitude. LV-GLS was moderately impaired, particularly at basal and mid-level, with apical sparing. LV-GLS left ventricular-global longitudinal strain.

On TTE, we observed that MAD presence was associated with a narrow A-P thoracic diameter (A-P magnitude = 11 cm), measured as the distance between the true apex of the sector and the posterior wall of the descending aorta. During conventional TTE examination, echocardiographic movies from each of the three apical views (four-chamber, two-chamber, and three-chamber) were acquired for the subsequent offline analysis of left ventricular global longitudinal strain (LV-GLS) by speckle tracking echocardiography (STE). LV-GLS was moderately impaired in comparison to the accepted reference values [31]. Segmental analysis of LV longitudinal strain showed a significant attenuation of basal and mid longitudinal strain, particularly at the level of the LV inferolateral segments, whereas the apical longitudinal strain was normal (apical sparing pattern) (Figure 3).

**Figure 4:**
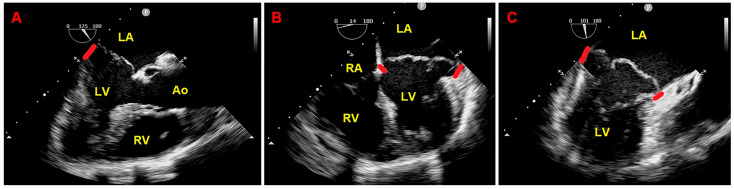
Transesophageal echocardiography. MAD assessment at end-systole from the mid-esophageal three-chamber view (**A**), four-chamber view (**B**), and two-chamber view (**C**). The bold red line indicates the MAD distance. Ao, aorta; LA; left atrium; LV, left ventricle; RA, right atrium; RV, right ventricle. (**A**) is reproduced from the ref. [4].

**Figure 5:**
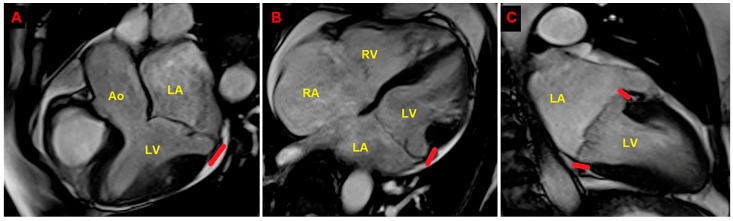
Cardiac magnetic resonance. MAD assessment at end-systole from the three-chamber view (**A**), four-chamber view (**B**), and two-chamber view (**C**). The bold red line indicates the MAD distance. Ao, aorta; LA; left atrium; LV, left ventricle; RA, right atrium; RV, right ventricle. (**A**) is reproduced from the ref. [4].

**Figure 6:**
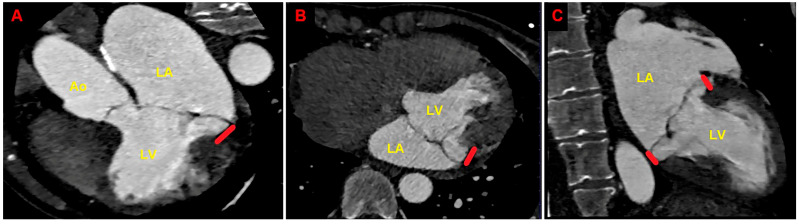
Cardiac computed tomography angiography. MAD assessment at end-systole from the multiplanar reconstructed three-chamber view (**A**), four-chamber view (**B**), and two-chamber view (**C**). The bold red line indicates the MAD distance. Ao, aorta; LA; left atrium; LV, left ventricle. (**A**) is reproduced from the ref. [4].

Due to the echocardiographic detection of wide MAD distance and MV floppy degeneration with moderate MR, the patient underwent a comprehensive diagnostic study of TEE, CMR, and CCTA. Transesophageal examination (Figure 4), CMR (Figure 5), and CCTA (Figure 6) confirmed the bileaflet floppy MVP, the circumferential extension of MAD, and the moderate degree of MR. All the imaging techniques were concordant with the MAD presence and its extent.

CMR excluded areas of focal late gadolinium enhancement (LGE). CCTA showed normal coronary arteries.

**Figure 7:**
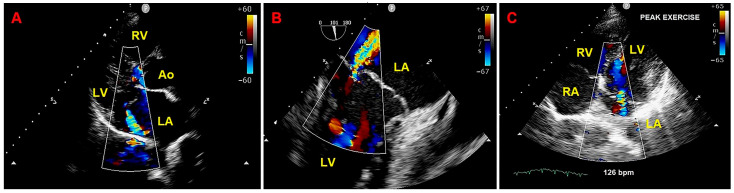
Moderate mitral regurgitation detected on resting transthoracic echocardiography from the parasternal long-axis view (**A**), on transesophageal echocardiography from the bicommissural view (**B**), and on exercise stress echocardiography from the apical four-chamber view recorded at peak exercise (**C**). Ao, aorta; LA; left atrium; LV left ventricle; RA, right atrium; RV, right ventricle.

Given that the patient was symptomatic for exercise-induced dyspnea and that we detected a moderate MR due to MVP on both TTE and TEE, she also underwent ESE. On ESE, the patient performed a maximal physical exercise (by achieving 85% of maximal age-predicted heart rate), the moderate MR did not show any significant modification in comparison to resting conditions (Figure 7), the pulmonary hemodynamics was normal (estimated peak exercise sPAP = 45 mmHg), the patient showed a good exercise tolerance and did not manifest palpitations.

**Figure 8:**
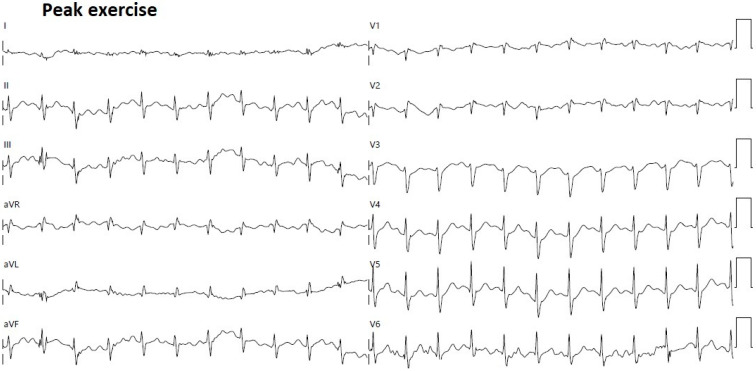
12-lead ECG obtained at peak exercise during exercise stress echocardiography. ECG, electrocardiogram.

Moreover, the electrocardiographic monitoring during ESE excluded the occurrence of complex VAs or significant ST-T abnormalities (Figure 8).

The aforementioned multi-instrumental evaluation allowed the detection of a potential benign phenotype of MAD, associated with a concave-shaped chest wall conformation (MHI > 2.5) and/or a narrow A-P thoracic diameter (<13.5 cm) [30], with a moderate and non-hemodynamically significant MR and low arrhythmic profile.

## 3. Discussion

### 3.1. The Role of CMR in MAD Assessment

CMR is actually considered the reference imaging modality for detecting MAD [3,32]. Due to its high signal-to-noise ratio, excellent blood-myocardium contrast-to-noise ratio, and reproducibility, this imaging modality allows us to accurately characterize the MAD presence and extent [33]. CMR is more sensitive than TTE for detecting small MAD <4 mm and for evaluating the whole circumferential extent of MAD [15]. Indeed, CMR has an incremental capacity to visualize the posterior MVA and its detachment from the LV wall over traditional TTE [34]. MAD causes a typical systolic curling motion of the basal inferolateral LV wall. Both MAD and systolic curling motion are associated with annular hypermobility and paradoxical systolic expansion, which can be appreciated on cine CMR images [9]. Excessive mobility of the mitral apparatus related to MVP and MAD can induce an abnormal mechanical stress/stretch on the basal inferolateral LV wall and/or the papillary muscles, possibly leading to the development of hypertrophy and fibrosis in these regions [9,12,35]. The greater the MAD extent, the higher the potential arrhythmic risk. With this regard, a MAD length >8.5 mm has been found to predict an increased risk of non-sustained ventricular tachycardia [8]. LGE sequences allow us to detect and precisely quantify these areas of myocardial fibrosis [12,36,37,38,39]. Additionally, CMR T1 mapping allows the non-invasive quantification of the myocardial extracellular volume (ECV), a marker of diffuse interstitial myocardial fibrosis [40,41]. Despite its advantages in the visualization and characterization of MAD, CMR has a lower spatial and temporal resolution than TTE. For this reason, CMR is less accurate than TTE in assessing leaflet thickness and calcifications. Moreover, CMR is less sensitive than TTE to visualize and quantify MR, may be less accurate in patients with arrhythmic disorders, and finally, is a time-consuming and not easily available and accessible technique [33].

### 3.2. The Role of Echocardiographic Techniques in MAD Assessment

The diagnostic accuracy of TTE and TEE in MAD identification is lower than that provided by CMR, particularly for MAD distance <4 mm, as recently demonstrated by Mantegazza V. et al. [15]. The lower detection rate of MAD by the echocardiographic techniques may be related to high acoustic impedance, with a suboptimal visualization of the posterior mitral valve annulus, or to the presence of posterior MVA calcification causing shadowing or reverberations. When compared to CMR, TTE and, to a lesser extent, TEE have a tendency to slightly overestimate the MAD distance [15]. The main advantages of echocardiography are its widespread availability, portable usage, low costs, and excellent capacity to assess MV morphology and evaluate the hemodynamic consequences of MR [42,43]. TEE may provide a better definition of MV anatomy and perivalvular structures. Given its semi-invasive nature, TEE is generally performed only in MAD patients with moderate-to-severe or severe MR before MV surgery [44].

ESE allows a dynamic evaluation of the MR degree associated with MVP and MAD [44]. A hemodynamically significant exercise-induced increase in mitral regurgitant volume and pulmonary pressures may have a prognostic relevance [45] and may aid in the decision-making process, particularly in patients with discordance between symptoms and regurgitation grade at rest [46,47].

Recent evidence indicates that a preliminary chest shape assessment, as noninvasively assessed by the MHI [30], might improve the prognostic risk stratification of symptomatic MVP patients with moderate MR. A MHI > 2.5 or a narrow A-P thoracic diameter (<13.5 cm), due to various degrees of anterior chest wall deformity, are commonly associated with a small left atrial size [48]. These individuals are commonly encountered in clinical practice and are frequently diagnosed by mild-to-moderate or moderate MR on conventional TTE. However, the severity of valvular heart disease may be overestimated in the presence of small cardiac chamber cavity sizes, particularly the left atrium as the receiving chamber. Indeed, both resting pulmonary hemodynamics and the peak-exercise sPAP are generally normal. Therefore, an apparently moderate MR disease may be more appropriately classified as mild-to-moderate or mild in those individuals with a narrow A-P thoracic diameter. Moreover, MVP patients with concave-shaped chest wall conformations have a low prevalence of adverse cardiovascular events over a mid-term follow-up [49].

### 3.3. The Role of CT in MAD Assessment

Due to its high spatial resolution, computed tomography (CT) may provide an accurate and highly reproducible evaluation of the mitral annulus and adjacent structures [50]. Literature data concerning the role of CT scans in MAD assessment are scanty. Putnam AJ et al. [29] assessed the MAD distance and circumferential extent in retrospectively ECG-gated cardiac CT with reconstruction of all phases throughout the R-R interval, whereas Tsianaka T et al. [51] performed a prospectively triggered high-pitch CT angiography study acquired at end-systole, demonstrating the reliability of CT for MAD identification and quantification. CT has the advantages of easy availability, short acquisition time, and the ability to view images in any desired plane using multiplanar reconstruction software. The risk of contrast-induced nephropathy and radiation exposure are the principal limitations of CT.

### 3.4. The Role of Chest Shape Conformation in MVP Individuals with MAD

Literature data indicate that MVP individuals are commonly found with various degrees of anterior chest wall deformity, ranging from mild concave-shaped chest wall conformation to severe forms of pectus excavatum [52,53]. The strong relationship between MVP and thoracic skeletal abnormalities (TSA) has been related to a defect in growth patterns at around the 5th to 6th week of gestation, affecting both the MV and the bony thorax [52,54]. Additionally, MVP is genetically associated with systemic connective tissue disorders, such as Marfan syndrome and Ehlers–Danlos syndrome [55]. Accordingly, the association between MVP and TSA is likely related to developmental or genetic factors.

Considering the strong association between MVP and TSA and the increased prevalence of MAD among MVP individuals [3,8,13,20,25,26,27,28], our study group hypothesized that MAD presence might be more frequently detected among individuals with a narrow A-P thoracic diameter and/or concave-shaped chest wall conformation. We previously demonstrated that compared to MAD- patients, MAD+ patients had significantly greater MHI, significantly shorter A-P thoracic diameter, significantly smaller cardiac chamber cavity sizes, and significantly lower magnitude of both longitudinal and circumferential myocardial strain parameters, particularly at the level of basal myocardial segments, with apical sparing [18]. The degree of strain impairment was strongly correlated to the degree of anterior chest wall deformity; the narrower the A-P thoracic diameter, the lower the myocardial strain magnitude in both longitudinal and circumferential directions, especially at the basal level. These findings would indicate the important role exerted by the anterior chest wall deformity in inducing an extrinsic compression of cardiac chambers, restricting LV kinetics of basal myocardial segments, and causing intraventricular and/or interventricular dyssynergies. The attenuation of myocardial strain parameters was detected in the presence of preserved biventricular systolic function, as assessed by LVEF and TAPSE, thus excluding intrinsic myocardial dysfunction. The absence of intrinsic cardiomyopathy is further supported by the preservation of the typical “base-to-apex gradient” (lowest to highest) of LV strain in MAD+ patients, whose impairment is characteristic of pathological states [56]. We also reported that MVP and MAD+ patients with MHI > 2.5 or A-P thoracic diameter < 13.5 cm had a low prevalence of complex VAs and a good outcome over a mid-term follow-up period [57].

### 3.5. Implications for Clinical Practice

Based on our previous findings and as demonstrated in the illustrated clinical case, we have hypothesized the existence of a cluster of patients with a benign phenotype of MAD, represented by individuals with a narrow A-P thoracic diameter (<13.5 cm) without LGE on CMR, without detectable complex VAs, with mild-to-moderate and non-hemodynamically significant MR due to MVP, with normal biventricular systolic function and with reduced basal longitudinal strain due to sternal compression.

A careful physical examination and comprehensive chest shape assessment should be routinely considered in MVP individuals. Both the non-radiological MHI and/or the echocardiographic A-P thoracic diameter may allow the cardiologists to identify, among the MVP individuals, those with an increased probability of nonmalignant MAD.

It could be then important to design high-powered multicentered prospective studies to evaluate the prognosis of MAD+ patients with a concave-shaped chest wall conformation over a mid-to-long term follow-up.

## 4. Conclusions

Not all MVP patients with MAD have an increased arrhythmogenic substrate.

MAD patients with a narrow A-P thoracic diameter may be found with non-hemodynamically significant MR, without LGE on CMR, and without detectable complex VAs.

The assessment of chest shape conformation might improve the prognostic risk stratification of MAD+ patients.

## Data Availability

Data extracted from the present case report will be publicly available on Zenodo (https://zenodo.org accessed on 15 February 2025), pending acceptance by the journal.
